# Household Air Pollution From Solid Cooking Fuel Combustion and Female Breast Cancer

**DOI:** 10.3389/fpubh.2021.677851

**Published:** 2021-08-04

**Authors:** Tanxin Liu, Ru Chen, Rongshou Zheng, Liming Li, Shengfeng Wang

**Affiliations:** ^1^Department of Epidemiology and Biostatistics, School of Public Health, Peking University Health Science Center, Beijing, China; ^2^National Cancer Center/National Clinical Research Center for Cancer/Cancer Hospital, Chinese Academy of Medical Sciences and Peking Union Medical College, Beijing, China

**Keywords:** household air pollution, breast cancer, cooking fuel, indoor air pollution, solid fuel

## Abstract

**Background:** Women bear a large share of disease burden caused by household air pollution due to their great involvement in domestic activities. Pollutant emissions are believed to vary by exposure patterns such as cooking and space heating. Little is known about the independent effect of solid cooking fuel combustion on breast cancer risk. We aimed to examine the association of indoor coal and wood combustion for cooking with breast cancer risk.

**Methods:** During June 2004–July 2008, participants aged 30–79 from 10 diverse regions across China were enrolled in the China Kadoorie Biobank. Primary cooking fuel use information in up to three residences was self-reported at baseline. Multivariable logistic regression models yielded adjusted odds ratios (ORs) and 95% confidence intervals (CIs).

**Results:** A total of 290,396 female participants aged 30–79 were included in the main analysis. Compared with long-term clean fuel users, the fully adjusted ORs were 2.07 (95%CI: 1.37–3.13) for long-term coal users, 1.12 (95% CI: 0.72–1.76) for long-term wood users, and 0.98 (95% CI: 0.55–1.74) for those who used mixed solid fuels to cook. Those who had switched from solid to clean fuels did not have an excess risk of breast cancer (OR: 0.88, 95%CI 0.71–1.10).

**Conclusion:** Long-term solid fuel combustion for cooking may increase the risk of breast cancer. The strength of association is stronger among coal users than wood users. Targeted interventions are needed to accelerate the access to clean and affordable energy.

## Introduction

Household air pollution (HAP) causes immense disease burden throughout the world. Around 3.8 million people died prematurely from illness attributed to HAP (1). Globally, “by far the most important direct health risk is the pollution caused by incomplete combustion of solid fuels for cooking, heating and lighting” (2). The adverse impacts from HAP are largely caused by energy poverty, especially in rural regions of the low-and middle-income countries (LMICs) where some residents lack access to affordable, clean energy such as electricity, biogas and gas (3). Instead, they rely on solid fuel collected from agricultural residues, hauled from kilometers away, or purchased at a low price to meet daily energy demand (3). According to the World Health Organization (3), solid fuel includes coal as well as biomass fuels (referring to renewable plant-based material such as wood, crop wastes and charcoal), providing heat and light during the process of combustion (4). Incomplete combustion of solid fuels produces high levels of HAP with a range of harmful pollutants, including particulate matter, sulfur oxides, nitrogen oxides, carbon monoxide, polycyclic aromatic hydrocarbons, formaldehyde, and dioxins, to name a few (5–9). In contrast, clean fuel mainly includes “electricity, liquefied petroleum gas (LPG), piped natural gas (PNG), biogas, solar and alcohol fuels”, which produces low levels of emissions of particulate matter, sulfur dioxide and other by-products of incomplete combustion when properly used (9). Although the past few years have witnessed a surge in technological innovation in the household energy sector, progress remains too slow to displace the polluting fuel combustion systems and thereby mitigate their health impacts. Based on the most recent global estimates, more than 2.7 billion people heavily relied on domestic solid fuels in 2015, including 450 million people in China (10).

Household air pollution from solid cooking fuel (notably coal and wood) has been categorized as a Group 2A carcinogen (11). Special attention should be placed to females who spend considerable amount of time in proximity to polluting sources due to their great involvement in daily cooking activity (4). Ambient air pollutants (e.g., particulate matter, polycyclic aromatic hydrocarbons) may cause tumor formation in breast and cervix uteri (12–14). Evidence for the relationship with household air pollutants remains scarce. Three previous studies have examined the indoor solid fuel combustion as a risk factor for breast cancer and yielded inconsistent result (15–17). Previous studies on this topic have mainly conducted in high-income countries and focused on wood burning (15, 16). However, in some coal-producing countries such as China and India, coal is considered as a domestic source of energy (11). There is a paucity of studies on the potential impact from indoor coal combustion for cooking. Furthermore, HAP from cooking and space heating are two different exposure patterns, which may have different influences on carcinogenesis. A stove might be kept going all day for heating in winter months (3). By contrast, cooking produces HAP several times per day with a shorter period (3, 18). Field measurement reported significantly lower emissions of pollutants from domestic solid fuel combustion during heating compared to those from cooking (19). Epidemiological evidence on HAP exposure from cooking and heating reported different associations with lung cancer (20). One previous CKB study on HAP from heating fuel use and breast cancer mortality did not find any evident relationship with breast cancer mortality (17). Little is known about the independent effect of cooking fuel use on female breast cancer risk. This study reported findings on the solid cooking fuel combustion with breast cancer risk among 290,396 females.

## Methods

### Study Population

We used the baseline data from China Kadoorie Biobank (CKB) (21). It was initially set up to recruit 500,000 permanent residents aged 35–74 years without a known disability in five rural and five urban regions (100,000 for each region) (Supplementary Figure 1). From June 2004 to July 2008, 512,891 participants aged 30–79 years (302,510 females, 59.0%) completed the baseline survey. To encourage participation, we included 10,715 participants whose age was slightly outside the target range, resulting in the baseline age range 30–79 years. In 2008, ~4% of participants were randomly selected to attend the resurvey with repeated interviews. Details of this biobank have been described elsewhere (21, 22).

Registered participants went to the local assessment stations after signing the informed consent. Trained health staffs conducted a computer-assisted interview with participants to collect a set of information, including demographics, lifestyle behaviors, and medical history via a standard electronic questionnaire. All participants also underwent physical measurements and a 10 ml blood sample collection. Ethical approval of CKB was obtained from the Ethical Review Committee of the Chinese Center for Disease Control and Prevention and the Oxford Tropical Research Ethics Committee.

### Assessment of Exposure and Outcome

Participants were asked to recall their cooking frequency, type of cooking fuels and ownership of ventilated stoves for up to three most recent residences (each lived at least 1 year), and duration (in years) in each residence. Participants were asked, “In your present & two previous houses, how often did you cook at home?” Participants chose from the options of daily, weekly, monthly, rarely/never, no cooking facility (23). For those who cooked at least monthly, we further asked their primary cooking fuel which they used most frequently at each residence (coal, wood, gas, electricity, other unspecified). Solid fuels included coal and wood, whereas clean fuels included gas and electricity. Participants who reported having cooking facilities were asked the presence of chimney or extractor related to cooking stove(s) used (23). Participants cooking daily or weekly were considered as cooking regularly (23–25). Long-term exposure pattern was examined by classifying participants who cooked regularly into three groups: those who always used the same fuel in all residences (always solid, always clean), and those who used solid fuels in previous residence(s) and then used clean fuels in the present residence. Participants who always used solid fuels were further divided into three groups (always wood, always coal, a mixture of coal and wood). All participants were asked if a doctor told them that they had had cancers and the site of cancers. If participants suffered from more than one cancer, the one that occurred first was recorded. The cancer status was also confirmed by the hospital admission in the resurvey. We considered breast cancer (ICD-10: C50.42) as our primary outcome.

### Covariates

Covariates of potential interest comprised of demographic characteristics, lifestyle factors, household air pollution, reproductive history and family history, which were selected based on previous literature on this topic (15, 16, 26). The demographic variables included age (continuous variable), study region (urban, rural), education (no education, primary school, middle school, high school and above), occupation (unemployed/retired, agricultural worker, factory worker, non-manual worker), annual family income (<10,000, 10,000–34,999, ≥35,000 yuan), marital status (married, never married/widowed/separated/divorced). Lifestyle and HAP variables included current smoking status (not smoke/occasionally, daily/on most days), alcohol drinking (never/rarely, occasionally/at certain season, monthly/weekly), body mass index (BMI) (continuous variable), environmental tobacco smoke exposure (ETS) (never/occasionally, 1–5 days a week, daily) and ownership of stove ventilation (all stoves, not all, none). Reproductive history included age at menopause (premenopausal, menopause age <50, menopause age ≥50), parity (0, 1, 2, ≥3), use of oral contraceptive pills (never, ever). We included physical activity levels (metabolic equivalent of task, hours/day), family history of cancer (presence or absence) and consumption of preserved vegetables (daily/4–6 days per week, 1–3 days per week, monthly, never/rarely) in our sensitivity analysis.

### Statistical Analyses

We restricted our analyses to females (*n* = 302,510) and excluded 2,238 participants who did not report cooking information at three residences or used other unspecified fuels, leaving 300,272 for baseline characteristics estimation. We further excluded participants who did not cook regularly at three residences (*n* = 8,839, 2.9%) and those with fluctuating exposure condition (using clean fuel at the first residence, solid fuel at the second residence and clean fuel again at the third residence) (*n* = 1,037, 0.03%). Finally, a total of 290,396 participants were included in the main analyses.

Adjusted values of baseline characteristics by cooking fuel category were presented, with adjustment for age and region where appropriate. We adopted multivariable logistic regressions to estimate odds ratios (ORs) of breast cancer. Model 1 was adjusted for age and study region (18). Model 2 adjustment included all demographic variables (age, study region, education, occupation, annual family income, marital status) and lifestyle variables (smoking, alcohol consumption, BMI, ETS, and ownership of stove ventilation) (15). Model 3 adjustment included all above variables and reproductive history (age at menopause, parity, contraceptive use) (15). We considered clean fuel group as our reference (defined as using gas and/or electricity in all recalled residences) (18, 23, 25). We also calculated the duration of solid fuel exposure during the recall period by summing the number of years at three residences where solid fuel (coal or wood) was reported as the primary cooking fuel. Duration of exposure was classified into three groups: never, duration <25, duration ≥25. Linear trend was tested by modeling a continuous variable that was assigned the median year of duration for each participants' exposure category (27). Considering the biology of female breast cancer and HAP, we stratified the analysis by environmental tobacco smoke exposure, menopause status and contraceptive use, controlling for the same set of covariates as appropriate. The tests for interaction were performed using likelihood ratio test comparing models with and without the cross-product term.

Several sensitivity analyses were further performed. First, we additionally adjusted for potential covariates, including physical activity, family history of cancer and consumption of preserved vegetables. Second, we excluded participants who smoked daily/on most days; those who were exposed to environmental tobacco smoke daily or almost every day; those who were nulliparous; those who had ever used oral contraceptive pills. Third, we selected the lag period of 5 years and 10 years, discounting the exposure during this period. Finally, we explored the association of HAP from solid cooking fuel use with breast cancer mortality, using time in study as the time scale. All analyses were performed using Stata software 15.1 (StataCorp, TX, USA).

## Results

Of the 300,272 females [mean (SD) age 51.46 (10.48) years], 51.1 % always used solid cooking fuel and 18.0% always used clean fuel in all residences. Females who always used solid fuels tended to be older, more likely to live in rural region, less educated, more exposed to passive smoking, less likely to use oral contraceptive pills and had lower household income in comparison with clean fuel users (Table 1).

**Table 1:** Baseline characteristics by cooking fuel use (*n* = 300,272^a^).

	**Cooking fuel exposure**			
**Characteristics**	**Solid fuel**	**Clean fuel**	**Solid to clean fuel^**b**^**	**No cooking**
No. of participants, *n* (%)	152802 (51.1)	53875 (18.0)	83719 (27.9)	8839 (3.0)
Age at baseline (y)	53.0	46.2	52.8	45.5
BMI (kg/m^2^)	23.5	24.0	24.3	24.4
Physical activity (MET-h/d)	20.3	19.4	21.0	23.4
Rural (%)	91.4	10.2	20.8	46.5
Married (%)	89.3	87.1	89.4	88.7
Primary school and lower (%)	6.8	3.6	5.6	5.5
Income <10,000 (Yuan^c^/y) (%)	41.0	11.7	18.8	8.4
No occupation (%)	22.6	39.7	40.6	21.2
Tobacco smoking^d^ (%)	3.9	4.5	4.6	4.5
Alcohol drinking^e^ (%)	1.7	2.9	1.9	3.1
Family history of cancer (%)	16.2	16.9	17.8	16.1
Passive smoking^f^ (%)	87.1	74.9	83.9	75.3
Good ventilation^g^ (%)	11.7	27.2	8.2	15.5
Postmenopause^h^ (%)	82.9	78.8	82.6	78.5
Having live birth (%)	98.8	98.5	99.1	97.1
Contraceptive use^h^ (%)	5.5	12.6	16.6	20.6

*We used linear models (for continuous variables) or logistic models (for categorical variables) to estimate predicted probabilities adjusted for age and region as appropriate*.

*BMI, body mass index; MET-h/d, metabolic equivalent task-hours per day*.

a *Participants were classified as clean or solid fuel users if they reported only using clean or solid fuel as primary cooking fuel at all three residences. The no cooking group included people who did not cook, or rarely cooked, or did not have cooking facility at three residences. Few participants reported fluctuating exposure condition (using clean fuel at first residence, solid fuel at second residence, and then clean fuel again at the third residence) (n = 1,037, 0.3%), thus this group were not presented*.

b *“Solid to clean fuel” exposure was defined as using solid fuel (coal, wood) as the primary cooking fuel at previous residences and then used clean fuel at the current residence*.

c *10,000 Yuan = 1412.6688 US dollar*.

d *Tobacco smoking was defined as smoking tobacco daily or on most days*.

e *Alcohol drinking was defined as drinking any alcohol usually at least once a week*.

f *Passive smoking was defined as ever lived with smoker in the same house for at least 6 months*.

g *Good ventilation was defined as all stoves for cooking with a chimney or extractor, or had no stoves at three residences*.

h *Variables had forty-three missing values*.

We documented 551 participants diagnosed with breast cancer. Compared with long-term clean cooking fuel use, long-term coal combustion was associated with a higher risk of breast cancer (fully adjusted OR:2.07, 95%CI: 1.37–3.13) (Table 2). Fully adjusted ORs of breast cancer were 1.12 (95%CI: 0.72–1.76) for those who always used wood, and 0.98 (95% CI: 0.55–1.74) for those who used mixed solid fuels to cook [mean duration of exposure: 16 years]. Long-term solid cooking fuel combustion [mean duration of exposure: 30 years] appeared to confer a higher risk of breast cancer, albeit not significant (fully adjusted OR,1.19 (95%CI: 0.84–1.67). There was no elevated cancer risk among women who had switched into clean fuels [mean duration of exposure: 18 years]. No evident relationship was observed between solid fuel use and breast cancer risk.

**Table 2:** Association of cooking fuel use with breast cancer risk among 290,396 participants^a^.

	**No. of participants at baseline, *n***	**Cases, *n***	**Model 1**	**Model 2**	**Model 3**
**Pattern of fuel use**					
Always clean fuel (reference)	53875	148	ref	ref	ref
Solid to clean fuel	83719	243	0.92 (0.74–1.14)	0.98 (0.79–1.22)	0.88 (0.71–1.10)
Always solid fuel	152802	160	0.80 (0.59–1.07)	1.19 (0.86–1.66)	1.19 (0.84–1.67)
Solid cooking fuel type^b^					
Always coal	56835	83	1.48 (1.01–2.17)	1.81 (1.22–2.67)	2.07 (1.37–3.13)
Always wood	65956	56	0.60 (0.42–0.87)	1.12 (0.72–1.74)	1.12 (0.72–1.76)
A mixture of coal and wood	30011	21	0.69 (0.40–1.19)	0.94 (0.53–1.65)	0.98 (0.55–1.74)
Duration of solid fuel exposure (y)^**c**^					
Never^d^	53875	148	ref	ref	ref
Duration <25	115971	252	1.01 (0.82–1.25)	1.07 (0.87–1.33)	1.02 (0.82–1.27)
Duration ≥25	120550	151	0.65 (0.50–0.85)	0.82 (0.63–1.08)	0.78 (0.59–1.03)
*P* for trend			0.0013	0.1561	0.0849

*Model 1 was adjusted for age and region. Model 2 was additionally adjusted for education, occupation, marital status, household income, body mass index (BMI), smoking status and alcohol consumption, environmental tobacco smoke and stoves with ventilation. Model 3 was further adjusted for age at menopause, parity and use of oral contraceptive pills*.

*OR, odds ratio; CI, confidence interval*.

a *Few people did not cook at three residences (n = 8,839, 2.9 %) or had switched from solid to clean fuels (n = 1,037, 0.3%), thus were excluded from the models. Clean fuel group was considered as common reference group*.

b *Solid fuel group did not include those who had switched from solid to clean fuels (n = 83,719)*.

c *Duration was calculated by summing the number of years in each residence where solid fuel (coal, wood) were reported as the primary cooking fuel*.

d *The never group included those who used clean fuel at three residences*.

**Figure 1:**
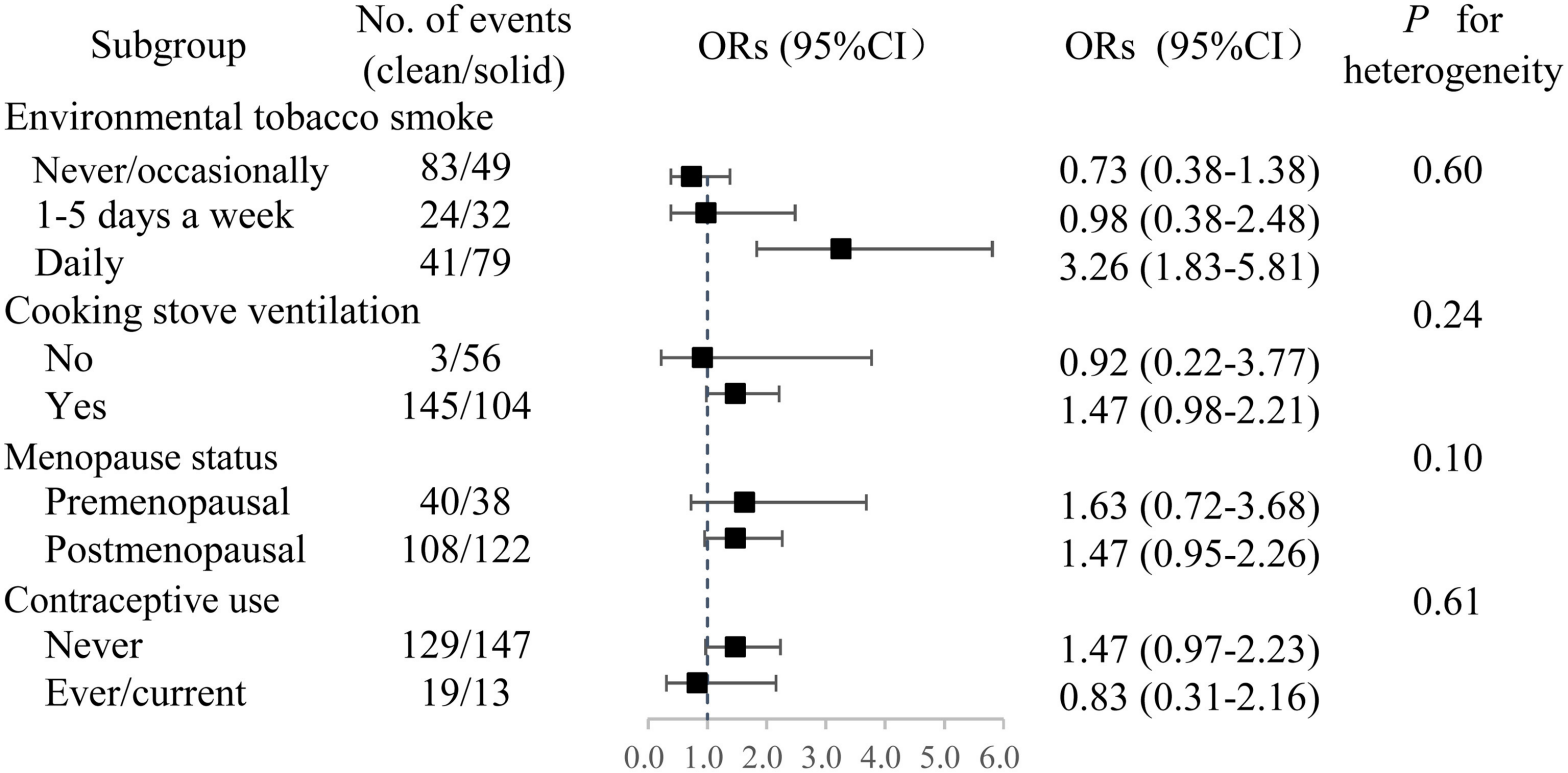
Adjusted ORs of breast cancer associated with long-term solid fuel use. Participants who had switched from solid to clean fuels or used a mixture of coal and wood were excluded. OR, odds ratio. CI, confidence interval.

There was no statistical effect measure modification by environmental tobacco smoking (ETS), cooking stove ventilation, menopausal status or contraceptive use (Figure 1). The strength of observed associations remained largely unchanged after excluding the mixed fuel users. The adjusted OR was somewhat stronger in females with daily ETS exposure (OR: 3.26, 95% CI: 1.83–5.81) than in those who got exposed to ETS 1–5 days per week (OR 0.98, 95% CI: 0.38–2.48) and in those who never/occasionally got exposed to ETS (OR 0.73, 95%CI: 0.38–1.38).

In the sensitivity analyses, the association of solid fuel exposure and breast cancer risk was unaltered after adjusting for potential confounders and excluding regular smokers, nulliparous women and those who had ever used oral contraceptive drugs (Supplementary Table 1). When 5-year or 10-year lag period was adopted, the strength of observed associations of two cancers appeared to be increased among long-term wood users and overall long-term solid fuel users, yielding significant results (Supplementary Tables 2, 3). We did not observe excess risk of breast cancer mortality, probably due to insufficient number of deaths (Supplementary Table 4).

## Discussion

In this study, we observed inconsistent associations of solid cooking fuel exposure with breast cancer risk. The adjusted ORs of breast cancer were not statistically significant among persistent solid fuel users in general (OR: 1.19, 0.84–1.67). In line with our finding, a case-control study of women on Long Island demonstrated no increased risk of breast cancer incidence in females who frequently burned wood in their home (16). However, when stratifying by type of solid fuel use, we observed a higher risk of breast cancer in persistent coal users (OR 2.07, 2.37–3.13) but not in persistent wood users. Apart from that, a prospective cohort study in the United States or Puerto Rico suggested that having indoor wood-burning stove/fireplace appeared to confer higher breast cancer risk (HR=1.11, 95%CI: 1.01–1.22) (15). Reports are inconsistent on which type of wood (synthetic or wood logs) can produce more polycyclic aromatic hydrocarbon (PAH) during domestic combustion (5, 6, 28). Previous association studies and risk assessment mainly focused on household wood combustion. The present study examined both wood and coal exposure and yielded inconsistent associations with breast cancer. Further prospective evidence is needed to elucidate the relationship of individual and combined effect of wood and coal exposure with breast cancer risk. Moreover, previous CKB study on heating fuel use did not observe excess risk of breast cancer mortality in any solid fuel groups (10). In contrast, this study focused on cooking fuel use and firstly suggested a positive association of long-term coal combustion for cooking with breast cancer risk. The strength of association remained largely unchanged in sensitivity analyses (Supplementary Tables 1–3). A possible explanation is that solid fuel combustion for cooking has a longer lifetime duration and thus provides higher cumulative inhaled pollutants compared to solid fuel combustion for heating (18). HAP from heating is a seasonal exposure during winter months while HAP from cooking is a regular exposure in this study since we included long-term solid fuel users who cooked daily or weekly in each residence lived at least 1 year. Differences in study design and covariates adjustment may also lead to different findings in two CKB studies. The association of HAP from different domestic activities (e.g., cooking and heating) with breast cancer risk needs future research to elucidate.

We observed no elevated breast cancer risk among women who had ceased using solid fuels. The point estimate of risk was lower in those who had switched from solid to clean fuels than long-term solid fuel users [OR]. Those who had ceased using solid fuels may get less exposed to solid fuel burning than long-term solid fuel users [duration in years: median (IQR): 16 (9–25) vs. 30 (21–41)]. Previous CKB study has demonstrated that the excess risk of all-cause mortality decreased by more than 60% in 5 years after cessation of indoor solid fuel burning (29). The present study further reported the health impact of cession from solid fuels on breast cancer risk. On the global basis, females bear a large share of disease burden caused by HAP due to their domestic roles (3). Our findings may have unique implications on females and suggest the reduction of solid fuel use for cooking. Targeted efforts are needed to accelerate the promotion of clean fuel production facilities and distribution networks.

The association between household air pollution and breast cancer is biologically plausible. Incomplete combustion of solid fuels releases many pollutants to the indoor and outdoor air, such as carbon monoxide, particulate matter, carcinogenic polycyclic aromatic hydrocarbons (PAHs) (5–9). Of all these pollutants, PAHs have been widely investigated and classified as carcinogenic to humans (IARC Group1) (30). About 60.5% of the global total PAH emissions were from combustion of biomass fuels including wood and crop residues (31). In China, coal and biomass fuel combustion are two major emission activates of PAHs, accounting for roughly 20 and 60%, respectively (32). The field measurements showed that the total emission factors (EFs) of 28 PAHs from solid fuel combustion during a regular cooking period ranged from 20.7 to 535 mg/kg (33). EFs of PAHs varied from several mg/kg for wood fuels to about 200mg/kg for bituminous coal, a dirty fuel burned in domestic stoves in rural China due to low cost (34). Different emission profiles between coal and biomass combustion were also observed for predominant individual PAHs including benzo[a]pyrene(BaP), pyrene (PYR), perylene (PER), Benzo[e]pyrene(BeP) and dibenzo[a,l]pyrene (DBalP) (33). Experimental evidence has confirmed that PAH metabolites can react with DNA and form PAH-DNA adducts, which leads to mutations of cancer related-genes and cell death (35–37). Potential carcinogenic pathways include sister chromatid exchange, mutations in TP53 as well as DNA methylation (26, 38, 39). BaP, a marker of carcinogenic potency of PAH mixture and an endocrine-disrupting pollutant, was associated with increased risk of breast cancer in a French cohort (13, 40).

Persistent coal users had a higher risk of breast cancer than persistent wood users. We cannot directly compare our estimate for coal exposure with prior studies. To our knowledge, this is the first study which reports the association of breast cancer with coal combustion for cooking. Our results should be interpreted with caution due to relatively small number of cases. Given the sample sizes in the subgroups, we have sufficient power for coal combustion analysis (approximately 100%) but not for wood combustion (<50%). Although the play of chance cannot be ruled out, our analysis may suggest that pollutants from coal combustion could have more hazardous effect on breast cancer development than those from wood combustion. Different fuel properties and environmental condition contributes to the different formation and changes of trace organics emitted from combustion which may have adverse effects on breast carcinogenesis (41). Results from a previous field emission test study revealed that there was a statistically positive relationship between PAH derivatives and corresponding parent PAHs in emissions from coal combustion, but insignificant relationships for those from wood burning (41). PAHs exposure could be ubiquitous and concurrent multiple indoor sources of PAHs were associated with a 30–50% increase in breast cancer risk (28). Similarly, PAH profiles from inhalation and digestion could be modifiable risk factors (28). Further studies are warranted to monitor the multiple sources of PAH emissions between coal and wood combustion for cooking and elucidate their association with breast cancer.

The chief strengths of this study include the large number of cooking fuel users (in particular for coal users), geographical diversity and completeness of data collection. Moreover, to discount the exposure that is thought irrelevant to the outcome, we conducted sensitivity analyses and selected 5-year or 10-year lag period. Our study has several limitations as well. First, the cross-sectional design of this study precludes a causal inference between solid fuel exposure and risk of breast cancer. Further prospective studies are needed to confirm the causal relationship. Second, like other CKB studies, recall-bias is possible because of the self-reported nature of the baseline survey. Nevertheless, about 78% of the participants in the resurvey reported the same type of cooking fuel as in the baseline survey, and the kappa value for cooking information was acceptable (0.6) (42). The physician-diagnosed cancer history was also confirmed by hospital admission information in the resurvey. Third, although self-reported primary cooking fuel has been adopted as a practical proxy of HAP in many studies, it remains an inherently limited indicator (3). It is possible that secondary fuel exposure and pollutants from neighborhood also contribute to the HAP. Primary fuel use represents a compromise which balances imperative of capturing detailed information on HAP with the pragmatic considerations such as feasibility of conducting surveys and eliciting reliable information from participants. Fourth, we did not account for ambient air pollution that might contribute to breast cancer risk. Since CKB public database did not disclose home address due to privacy protection, GIS method (grid-based method) cannot be used to locate and control for ambient air pollutants. However, CKB disclosed the province where study participants were resided in, and in each province the study participants were all located in the same community or village. Although we could not obtain ambient air pollution data, we adjusted for study region in all models and assumed a similar pattern of ambient air pollution exposure from the same region (29). We expected this strategy could somehow account for residual confounding from ambient air pollution (29). Finally, CKB project does not include histotype or genetic information.

## Conclusion

Household air pollution from solid cooking fuel combustion may elevate the risk of female breast cancer. The strength of the association is higher in long-term coal users than in long-term wood users. This study may have global implications as many countries are in the transition to clean energy. Efforts to disseminate clean and affordable alternatives (electricity and gas) are gaining momentum in LMICs (3). Adoption of sustainable clean energy solutions hinges on improved understanding of gender dynamics of household energy use and sex-specific health impacts (3). Gender-responsive interventions which taking into account the gender roles in household energy acquisition and uses are required. More evidence on health impacts on females is needed for implementation of policies to promote health, as females are often the primary cooking fuel users and the ones who benefit most from transition to clean cooking fuels (3).

## Data Availability Statement

The raw data supporting the conclusions of this article will be made available by the authors, without undue reservation.

## Ethics Statement

The studies involving human participants were reviewed and approved by Ethical Review Committee of the Chinese Center for Disease Control and Prevention and the Oxford Tropical Research Ethics Committee. The patients/participants provided their written informed consent to participate in this study.

## Author Contributions

TL and SW designed the study. SW acquired the data. TL analyzed and drafted the manuscript. All authors contributed to the interpretation of data and revised the article. All authors read and approved the final article.

## Conflict of Interest

The authors declare that the research was conducted in the absence of any commercial or financial relationships that could be construed as a potential conflict of interest.

## Publisher's Note

All claims expressed in this article are solely those of the authors and do not necessarily represent those of their affiliated organizations, or those of the publisher, the editors and the reviewers. Any product that may be evaluated in this article, or claim that may be made by its manufacturer, is not guaranteed or endorsed by the publisher.

## Abbreviations

HAP: household air pollution
PAHs: Polycyclic aromatic hydrocarbons
95%CI: confidence interval
HRs: hazard ratios
IQR: interquartile range
SD: standard deviation.
