# Supplementary material for: Household Air Pollution From Solid Cooking Fuel Combustion and Female Breast Cancer
Source: Front Public Health. 2021 Aug 4;9:677851. doi: 10.3389/fpubh.2021.677851 (PMC8371394; doi:10.3389/fpubh.2021.677851)
Supplement: Supplementary file 1 [file Table_1.DOCX]

# Supplementary materials

# Household air pollution from cooking fuel combustion and female breast cancer

Tanxin Liu^1^, Ru Chen^2^, Rongshou Zheng^2^, Liming Li^1^, Shengfeng Wang^1,^*

1. Department of Epidemiology and Biostatistics, School of Public Health, Peking University Health Science Center, Beijing, China;

2. National Cancer Center/National Clinical Research Center for Cancer/Cancer Hospital, Chinese Academy of Medical Sciences and Peking Union Medical College, Beijing, China

*Address correspondence to: Shengfeng Wang, Email: shengfeng1984@126.com

Email addresses:

| Tanxin Liu | tliu76@jh.edu |
| --- | --- |
| Ru Chen | chenru1900@163.com |
| Rongshou Zheng | zhengrongshou@cicams.ac.cn |
| Liming Li | lmlee@bjmu.edu.cn |
| Shengfeng Wang | shengfeng1984@126.com |

# Table of Contents

[Supplementary materials 1](#_Toc74776684)

[Household air pollution from cooking fuel combustion and female breast cancer 1](#_Toc74776685)

[Table of Contents 2](#_Toc74776686)

[SFigure 1. Locations of the 10 survey regions of China Kadoorie Biobank. 3](#_Toc74776687)

[sTable 1. Sensitivity analysis for adjusted odds ratios of breast risk by long-term cooking fuel use^a^. 4](#_Toc74776688)

[sTable 2. Association of cooking fuel exposure category with breast cancer risk (OR and 95% CI) among 266,859 participants (5-year lag period) ^a^. 5](#_Toc74776689)

[sTable 3. Association of cooking fuel exposure category with breast cancer (OR and 95% CI) among 262,905 participants (10-year lag period) ^a^. 6](#_Toc74776690)

[sTable 4. Association of cooking fuel use with breast cancer mortality among 290,396 participants^a^ 7](#_Toc74776691)

[References for the supplementary material 8](#_Toc74776692)

### SFigure 1. Locations of the 10 survey regions of China Kadoorie Biobank.

Adopted from Chan et al. 2019; Chen et al. 2011.

Open circles indicate rural regions and solid circles indicate urban regions. Number of participants in each study region is shown in brackets.

### sTable 1. Sensitivity analysis for adjusted odds ratios of breast risk by long-term cooking fuel use^a^.

|  |  | Pattern of fuel use | | | Cooking fuel type | | |
| --- | --- | --- | --- | --- | --- | --- | --- |
|  | Number of events | Always clean fuel | Solid to clean fuel | Always solid fuel | Always coal | Always wood | Mix of coal and wood |
| Main analysis | 551 | ref. | 0.88 (0.71-1.10) | 1.19 (0.84-1.67) | **2.07 (1.37-3.13)** | 1.12 (0.72-1.76) | 0.98 (0.55-1.74) |
| Additional adjustment^b^ | 551 | ref. | 0.92 (0.73-1.15) | 1.21 (0.86-1.71) | **1.92 (1.26-2.92)** | 1.25 (0.79-1.97) | 1.04 (0.58-1.86) |
| Excluding regular smokers | 521 | ref. | 0.89 (0.71-1.12) | 1.20 (0.84-1.71) | **2.13 (1.39-3.26)** | 1.19 (0.75-1.88) | 1.05 (0.58-1.89) |
| Excluding nulliparous women | 540 | ref. | 0.89 (0.71-1.12) | 1.21 (0.85-1.70) | **2.17 (1.44-3.31)** | 1.11 (0.70-1.75) | 1.04 (0.58-1.85) |
| Excluding Contraceptives users | 486 | ref. | 0.94 (0.74-1.19) | 1.16 (0.80-1.69) | **2.31 (1.48-3.59)** | 1.15 (0.70-1.88) | 1.02 (0.55-1.90) |

^a^Odds ratios (95%CI) were derived from fully adjusted model (Model 3).

^b^Odds ratios were further adjusted for physical activity levels (metabolic equivalent of task, hours/day), family history of cancer (presence or absence) and consumption of preserved vegetables (daily/4-6 days per week, 1-3 days per week, monthly, never/rarely)

### sTable 2. Association of cooking fuel exposure category with breast cancer risk (OR and 95% CI) among 266,859 participants (5-year lag period) ^a^.

|  | Pattern of fuel use | | | Cooking fuel type | | |
| --- | --- | --- | --- | --- | --- | --- |
|  | Always clean fuel | Solid to clean fuel | Always solid fuel | Always coal | Always wood | Mix of coal and wood |
| Number of participants at baseline, n | 48,065 | 65,944 | 152,850 | 56,826 | 69,872 | 26,152 |
| Cases, n | 120 | 150 | 234 | 132 | 85 | 17 |
| Model 1 | Reference | 0.81 (0.63-1.03) | 1.68(1.30-2.18) | 3.13 (2.33-4.19) | 1.09 (0.80-1.48) | 1.00 (0.58-1.74) |
| Model 2 | Reference | 0.85 (0.66-1.09) | 2.78 (2.11-3.65) | 3.65 (2.71-4.90) | 2.14 (1.49-3.07) | 1.34 (0.77-2.36) |
| Model 3 | Reference | 0.80 (0.62-1.04) | 2.70 (2.04-3.57) | 3.66 (2.70-4.97) | 1.98 (1.37-2.86) | 1.24 (0.70-2.19) |
| Model 4 | Reference | 0.75 (0.58-0.97) | **2.68 (2.01-3.57)** | **3.75 (2.76-5.11)** | **1.95 (1.33-2.86)** | 1.22 (0.69-2.16) |

Note: ^a^We assumed a lag period of 5 years in the analyses. Model 1 was adjusted for age and region. Model 2 was additionally adjusted for education, occupation, marital status, household income, body mass index (BMI), smoking status and alcohol consumption. Model 3 was further adjusted for environmental tobacco smoke and stoves with ventilation. Model 4 was further adjusted for age at menopause, parity, use of oral contraceptives.

OR, odds ratio. CI, confidence interval

### sTable 3. Association of cooking fuel exposure category with breast cancer (OR and 95% CI) among 262,905 participants (10-year lag period) ^a^.

|  | Pattern of fuel use | | | Cooking fuel type^a^ | | |
| --- | --- | --- | --- | --- | --- | --- |
|  | Always clean fuel (reference) | Solid to clean fuel | Always solid fuel | Always coal | Always wood | Mix of coal and wood |
| No. of participants at baseline, n | 53,412 | 63,154 | 146,339 | 58,567 | 64,762 | 23,010 |
| Cases, n | 128 | 140 | 196 | 110 | 74 | 12 |
| Model 1 | Reference | 0.77 (0.60-0.99) | 1.50 (1.15-1.96) | 2.62 (1.92-3.56) | 1.06 (0.77-1.45) | 0.82 (0.43-1.56) |
| Model 2 | Reference | 0.80 (0.63-1.03) | 2.33 (1.76-3.10) | 3.03 (2.22-4.13) | 2.09 (1.44-3.03) | 1.08(0.57-2.07) |
| Model 3 | Reference | 0.75(0.58-0.97) | 2.27 (1.70-3.03) | 3.02 (2.20-4.14) | 1.93 (1.33-2.81) | 1.00 (0.52-1.92) |
| Model 4 | Reference | 0.71 (0.55-0.92) | **2.24 (1.67-3.01)** | **3.23 (2.34-4.44)** | **1.82 (1.23-2.69)** | 1.00 (0.52-1.93) |

Note: ^a^ In this analysis, we assumed a lag period of 10 years in the analyses. Model 1 was adjusted for age and region. Model 2 was additionally adjusted for education, occupation, marital status, household income, body mass index (BMI), smoking status and alcohol consumption. Model 3 was further adjusted for environmental tobacco smoke and stoves with ventilation. Model 4 was further adjusted for age at menopause, parity, use of oral contraceptives. we excluded participants who had switched from solid to clean fuels (n=63,154). Clean fuel group was considered as the common reference group.

OR, odds ratio. CI, confidence interval.

### sTable 4. Association of cooking fuel use with breast cancer mortality among 290,396 participants^a^

|  | No. of participants at baseline, n | Cases, n | Model 1 | Model 2 | Model 3 |
| --- | --- | --- | --- | --- | --- |
| Pattern of fuel use |  |  |  |  |  |
| Always clean fuel (reference) | 53875 | 49 | ref | ref | ref |
| Solid to clean fuel | 83719 | 104 | 1.17 (0.83-1.66) | 1.10 (0.76-1.57) | 1.14 (0.79-1.65) |
| Always solid fuel | 152802 | 116 | 0.91 (0.58-1.41) | 0.89 (0.54-1.46) | 0.96 (0.58-1.60) |
| Solid cooking fuel type ^b^ |  |  |  |  |  |
| Always coal | 56835 | 50 | 1.11 (0.63-1.96) | 0.92 (0.48-1.80) | 1.01 (0.52-1.99) |
| Always wood | 65956 | 45 | 0.76 (0.44-1.30) | 0.90 (0.48-1.71) | 0.99 (0.52-1.90) |
| Mix of coal and wood | 30011 | 21 | 0.82 (0.42-1.61) | 0.79 (0.38-1.64) | 0.86 (0.41-1.81) |
| Duration of solid fuel exposure (y)**^c^** |  |  |  |  |  |
| Never^d^ | 53875 | 49 | ref | ref | ref |
| Duration<25 | 115971 | 122 | 1.22 (0.86-1.72) | 1.38 (0.79-1.63) | 1.19 (0.83-1.72) |
| Duration≥25 | 120550 | 98 | 0.86 (0.57-1.28) | 0.83 (0.55-1.28) | 0.87 (0.56-1.34) |
| *P* for trend |  |  |  |  |  |

Note: Cox regression was used to estimate the hazard ratios (HRs) for breast cancer in association with cooking fuel use. We used age as the underlying time scale. Model 1 was adjusted for age and region. Model 2 was additionally adjusted for education, occupation, marital status, household income, body mass index (BMI), smoking status and alcohol consumption, environmental tobacco smoke and stoves with ventilation. Model 3 was further adjusted for age at menopause, parity and use of oral contraceptive pills.

HR, hazard ratio. CI, confidence interval

^a^ Few people did not cooking at three residences (n=8,839, 2.9 %) or had switched from solid to clean fuels (n= 1,037, 0.3%), thus were excluded from the models. Clean fuel group was considered as common reference group.

^b^ Solid fuel group did not include those who had switched from solid to clean fuels (n= 83,719).

^c^Duration was calculated by summing the number of years in each residence where solid fuel (coal, wood) were reported as the primary cooking fuel.

^d^The never group included those who used clean fuel at three residences.

### References for the supplementary material

Chan, K. H., Bennett, D. A., Kurmi, O. P., Yang, L., Chen, Y., Lv, J.,et al. China Kadoorie Biobank Study, G. (2019). Solid fuels for cooking and tobacco use and risk of major chronic liver disease mortality: a prospective cohort study of 0.5 million Chinese adults. *Int J Epidemiol*. doi:10.1093/ije/dyz216

Chen, Z., Chen, J., Collins, R., Guo, Y., Peto, R., Wu, F., et al. China Kadoorie Biobank, C. K. B. C. (2011). China Kadoorie Biobank of 0.5 million people: survey methods, baseline characteristics and long-term follow-up. *Int J Epidemiol, 40*(6), 1652-1666. doi:10.1093/ije/dyr120
